# Moving Toward and Through Trauma: Participant Experiences of Multi-Modal Motion-Assisted Memory Desensitization and Reconsolidation (3MDR)

**DOI:** 10.3389/fpsyt.2021.779829

**Published:** 2021-12-22

**Authors:** Tristin Hamilton, Lisa Burback, Lorraine Smith-MacDonald, Chelsea Jones, Matthew R. G. Brown, Cynthia Mikolas, Emily Tang, Kaitlin O'Toole, Priyanka Vergis, Anna Merino, Kyle Weiman, Eric H. G. J. M. Vermetten, Suzette Brémault-Phillips

**Affiliations:** ^1^Heroes in Mind, Advocacy and Research Consortium (HiMARC), Faculty of Rehabilitation Medicine, University of Alberta, Edmonton, AB, Canada; ^2^Department of Psychiatry, Faculty of Medicine and Dentistry, University of Alberta, Edmonton, AB, Canada; ^3^Department of Occupational Therapy, Faculty of Rehabilitation Medicine, University of Alberta, Edmonton, AB, Canada; ^4^Department of Psychiatry, Leiden University Medical Center, Leiden, Netherlands; ^5^Edmonton Operational Stress Injury Clinic, Alberta Health Services, Edmonton, AB, Canada; ^6^Department of Computing Science, Faculty of Science, University of Alberta, Edmonton, AB, Canada; ^7^Department of Psychiatry, ARQ National Psychotrauma Center, Diemen, Netherlands; ^8^Military Mental Health Care, Ministry of Defense, Utrecht, Netherlands

**Keywords:** 3MDR, PTSD, moral injury, military, veteran, trauma, virtual reality

## Abstract

**Introduction:** Military members and Veterans are at risk of developing combat-related, treatment-resistant posttraumatic stress disorder (TR-PTSD) and moral injury (MI). Conventional trauma-focused therapies (TFTs) have shown limited success. Novel interventions including Multi-modal Motion-assisted Memory Desensitization and Reconsolidation therapy (3MDR) may prove successful in treating TR-PTSD.

**Objective:** To qualitatively study the experiences of Canadian military members and Veterans with TR-PTSD who received the 3MDR intervention.

**Methods:** This study explored qualitative data from a larger mixed-method waitlist control trial testing the efficacy of 3MDR in military members and veterans. Qualitative data were recorded and collected from 3MDR sessions, session debriefings and follow-up interviews up to 6 months post-intervention; the data were then thematically analyzed.

**Results:** Three themes emerged from the data: (1) the participants' experiences with 3MDR; (2) perceived outcomes of 3MDR; and (3) keys to successful 3MDR treatment. Participants expressed that 3MDR provided an immersive environment, active engagement and empowerment. The role of the therapist as a coach and “fireteam partner” supports the participants' control over their therapy. The multi-modal nature of 3MDR, combining treadmill-walking toward self-selected trauma imagery with components of multiple conventional TFTs, was key to helping participants engage with and attribute new meaning to the memory of the traumatic experience.

**Discussion:** Preliminary thematic analysis of participant experiences of 3MDR indicate that 3MDR has potential as an effective intervention for combat-related TR-PTSD, with significant functional, well-being and relational improvements reported post-intervention.

**Conclusion:** Military members and Veterans are at risk of developing TR-PTSD, with worse outcomes than in civilians. Further research is needed into 3MDR and its use with other trauma-affected populations.

## Introduction

Military members (MMs) and Veterans are at risk of post-traumatic stress disorder (PTSD) due to increased exposure to highly traumatic and stressful events (1, 2). A complex condition, PTSD is associated with hypervigilance, impaired cognition, comorbid mood and anxiety disorders, and significant functional and relational sequelae (1). The incidence rates of military-related PTSD in Canada are approximately 11% for MMs and 16% for Veterans (3). MMs and Veterans are also at risk of developing moral injury (MI)—persistent distress that can develop from exposure to potentially morally injurious events (PMIEs) (4–6) including perpetrating, witnessing, or failing to prevent an act that transgresses core values and beliefs (7). Up to 67% of MMs are exposed to PMIEs during deployments. The sequelae of PMIE exposure have not yet been fully explored, though it is suggested that MIs might contribute to TR-PTSD (8, 9).

Conventional trauma-focused therapies (TFTs) for PTSD are currently offered to MMs and Veterans, including Cognitive Processing Therapy (CPT), Exposure therapy, and Eye-Movement Desensitization and Reprocessing (EMDR). These TFTs, however, have been found to be poorly tolerated by MMs and Veterans, have limited success and high dropout rates and result in no better outcomes than non-TFTs (10, 11). Pharmacological treatment options have equally been shown to be problematic, with only two medications being approved as first-line treatment (12). Kelmendi et al. (13) also noted that <30% of persons achieve full remission, and even then, approved pharmacological treatments often take weeks for therapeutic effect. While there is a common belief that the combination of TFTs and pharmacological options would improve treatment outcomes, there is limited research to support this belief (14, 15).

Recovery or improvement rates for these TFTs is estimated at 31%, with drop-out rates ranging from 25 to 48% (10–12, 14–17). Avoidance of trauma-related feelings and memories has been identified as a key factor contributing to poor response and drop-out (18–20). Even in treatment responders, PTSD symptoms often persist at or above diagnostic thresholds for PTSD, with approximately 60% retaining the diagnosis (21, 22). MMs and Veterans who do not experience a clinically significant reduction in symptoms following at least two evidence-based treatments are considered to have treatment-resistant PTSD (TR-PTSD) (23, 24). Given the comorbid incidence of TR-PTSD and MI, new treatment options are needed.

Multi-modal Motion-assisted Memory Desensitization and Reconsolidation (3MDR) is an exposure-based, virtual-reality (VR) supported intervention developed in the Netherlands that incorporates treadmill walking within an immersive virtual reality environment. Multi-sensory cues, combined with physical movement, helps patients access and engage with emotional/cognitive trauma networks (25), breaking through persistent cognitive avoidance (26, 27) and providing MMs and Veterans with TR-PTSD opportunities to engage in reconsolidation (i.e., integration of new information) (26). Recently published RCTs conducted with Veterans with TR-PTSD are contributing to the evidence-base regarding 3MDR's effectiveness (28, 29).

The 3MDR protocol used in this study consists of 8 sessions over an 8-week period, beginning with one to two pre-platform preparatory session(s) during which the patient selects and orders images related to traumatic experiences, as well as music both reminiscent of past trauma and supportive of reconnecting with the present. Participants were also familiarized with the virtual environment and 3MDR protocol in a preparatory session. Each of six platform sessions were 90-min therapy sessions in the virtual reality environment (VRE). During a 60-min platform phase, the patient dons a safety harness and walks continuously on a treadmill while accompanied by a therapist. The participant warms up by listening to music that connects them to their traumatic experiences and then, during each of seven 3- to 5-min cycles, walks down a virtual “hallway” toward a trauma-related image. The participant describes each image, physical sensations, and feelings, and identifies, with help from the therapist, descriptive words/phrases which are then projected on the image, which participants repeat aloud. For a duration of 30 s, the participant then reads out numbers as they appear on a ball, which oscillates horizontally in the foreground of the image and words. After the seventh cycle, music is played for a cool-down period, helping the participant re-connect to the present. Each session concludes with a post-platform phase and debrief, which provides a place for the therapist and participant to reconsolidate learnings made on the platform (i.e., what experience stands out or brought new meaning for the participant) and conduct a mental wellness check and/or self-care plan. A post-intervention session occurred where an in-depth, semi-structured qualitative interview with quantitative questionnaires were conducted (26, 30). The protocol described here is slightly modified from the 10-session protocol that is currently used in the Netherlands.

### Objectives

This manuscript aims to present preliminary qualitative results examining participant experiences of 3MDR. This study is part of an ongoing larger mixed-methods waitlist control trial study examining the effectiveness of 3MDR with trauma-affected populations.

## Methods

### Study Design

The larger study consists of a mixed-methods waitlist control trial, capturing quantitative (self-reported validated questionnaires), biological, and qualitative data (see 29 for details of the 3MDR protocol). Given the rich data collected to date, the research team felt it was appropriate to present the quantitative and qualitative findings independently. The results reported here are based on the qualitative components of the study. Studies exploring the quantitative and biological data will be forthcoming. Ethical approval from the University of Alberta Research Ethics Board (Pro00084466) and Canadian Armed Forces Surgeon General Endorsement (E2019-02-250-003-0003) were received prior to study initiation.

### Data Collection and Analysis

Qualitative data consisted of (1) audio/video-recordings and chart notes collected during 3MDR sessions; (2) post-session debriefings, during which the therapist and participant reconsolidated learnings and made a self-care or safety plan; and (3) post-intervention interviews at baseline (T1), treatment sessions (T2.1–T2.6), and follow-up interviews at 1 week (T3), 1 month (T4), 3 months (T5) and 6 months (T6) post-intervention. Post-intervention interview questions included: “What intervention was the most/least helpful/impactful/effective?”; “How might the intervention be improved?”; “Has your participation in 3MDR impacted you in the way you had hoped/anticipated? Has it impacted your quality of life, relationships, social integration/engagement, housing, health, employment and other meaningful activity, finances, or life skills?”

Qualitative data were transcribed and thematically analyzed using Braun and Clarke's method (31, 32). Research team members at arm's length from the delivery of 3MDR independently performed inductive and deductive thematic analysis. In particular, two teams of three were created, with one team conducting deductive coding through pre-established a priori codes, while the other team looked specifically for inductive codes. Each team independently reviewed the qualitative data, and once completed, developed a coding structure to help organize their thoughts. At this stage, both teams reconvened at weekly team meetings to allow for rich discussion and comparison between the two teams, ensuring conformability and inter-rater reliability as well as bracketing of researcher bias (33). A larger team, including those who delivered the 3MDR intervention, supported a secondary round of deductive analysis and supported code verification, resolution of discrepancies, and determination of final themes with supporting quotes.

## Results

Findings include both demographic information and the qualitative thematic analysis results.

### Demographics

Participants (*n* = 13) included male (*n* = 12) and female (*n* = 1) Veterans (*n* = 10) and MMs (*n* = 3) ranging in age between 30.9 and 54.3 years (M = 44.7, SD 6.5) and having 5–10 (*n* = 3), 11–15 (*n* = 2) and 20+ (*n* = 8) years of service. All participants met the criteria for TR-PTSD (Table 1).

**Table 1:** Participant demographics.

**Sex (*n*; %)**	**Age (years)**	**Marital status**	**Employment status**
Female (*n* = 1; 8%)	Mean 44.7 ± 6.5 SD	Common-law (*n* = 2; 15%)	Unemployed (*n* = 6; 46%)
Male (*n* = 12; 92%)	Range: 30.9–54.3	Divorced (*n* = 1; 8%)	Employed (*n* = 7; 54%)
	30–39 (*n* = 3; 23%)	Married (*n* = 7; 54%)		
	40–49 (*n* = 7; 54%)	Separated (*n* = 1; 8%)		
	50–59 (*n* = 3; 23%)	Single (*n* = 2; 15%)		
**Active vs. Veteran**	**Enrollment Era**	**Rank**	**Element**	**Years of Service**
Active (*n* = 3; 23%)	1976–1990 (*n* = 2; 15%)	Junior NCM (*n* = 8; 62%)	Air (*n* = 2; 15%)	5–10 (*n* = 3; 23%)
Veteran (*n* = 10; 77%)	1991–2000 (*n* = 8; 62%)	Senior NCM (*n* = 4; 31%)	Land (*n* = 11; 85%)	11–15 (*n* = 2; 15%)
	2001–2015 (*n* = 3; 23%)	Unknown (*n* = 1; 8%)		20+ (*n* = 8; 62%)

### Qualitative Thematic Analysis

Qualitative thematic-analysis examined: (1) participants' experiences of 3MDR; (2) perceived outcomes following 3MDR; and (3) keys to successful 3MDR experiences. A discussion of each theme is followed by supporting quotes.

#### Theme 1: The Participant's Experience With 3MDR

3MDR participants reported high levels of engagement, and frequently described raw emotions and physiologic sensations as they explored traumatic memories. Sub-themes include: “This Feels Real”, “A Fireteam Partner” and “Breaking Through”.

##### “This Feels Real”: Safety in a Multi-Modal Immersive Environment

A combination of visual (photos), auditory (music), somatic (walking, attending to physical sensations), and verbal (description of events) cues created an immersive environment that challenged avoidance and enabled participants to connect with traumatic memories. “*[With 3MDR], you feel a lot more exposed for sure, a lot more raw. You're walking toward it—or rather, through it”* (P6;T2.1). Walking appeared to be reminiscent of marching during military service and contributed to the immersive nature of 3MDR. Walking also seemed to be both grounding and activating: “*I'm walking, I'm concentrating on what my body's feeling… I'm looking at photos I haven't looked at with that amount of intensity in fucking years”* (P7;T2.1). Preparation and repetition inherent in the 3MDR protocol were facilitative. “*Preparation is everything. [If] you know what is coming, it's not a shock; and if it's not a shock, we don't have to react”* (P2;T3). “*[A]s you get to know what to expect, it's a little [easier] to trust the process. It's not as overwhelming”* (P6;T2.8). The 3–5 min cycles focused their attention, such that participants were not simply “*go[ing] through the motions.”* Paced, brief exposures slowly increased their tolerance and confidence.

“*When I look at that picture, I put my boots on the edge and I was there... What I'm seeing there is not what you are seeing... I'm able to be there by standing there. That picture put me there.” (P11;T1)*

“*To see some of the images, and then to talk about them, and then go through the emotions and not be able to walk away or say ‘yeah I'm going to go to the washroom' or something like that, that was very important to be in it.” (P6;T5)*

“*In a typical therapy session where I'm sitting down, and the lights are down, and there's no stress and it's a safe spot and I can say whatever I feel like - well, if I don't want to talk about something, then I'm not going to talk about it… but with this… having to concentrate on so many different things, you don't have time to sit back and go ‘how do I not want to answer this?' It's just ‘how do I answer this? How do I actually feel?” (P7;T5)*

##### “A Fireteam Partner”: Figuring It Out Together

The relationship between the 3MDR therapist and participant exemplified a “fireteam partnership”—a relationship between reliable and compatible comrades, accountable to and for one another. The partnership promoted a trusting, safe, and non-judgmental environment and therapeutic alliance: “*To have somebody beside you the whole way, talking you through it, and [being] there for you is huge”* (P7;T5); “*We can calm down and figure out the next step instead of letting it all rip me down at once”* (P11;T3). Participants valued being able to tell their story in their own words and indicated that they felt comfortable with therapists asking probing questions, even while “*calling me on my bullshit—[T]hat's what I needed: people there to have my back”* (P6;T5). This partnership was so significant that some participants felt that any other therapist “*might not have been able to get the same results”* (P13;T4). The establishment and maintenance of the participant-therapist relationship, including consistency over the course of treatment, was foundational to the therapeutic process. Importantly, the fireteam partnership offered a different sense of connection and understanding.

“*That's what I needed (...) So you're just getting me to talk about shit that has fucked me up; there is no judgement.”* (P19;T3)

“*It's different [with 3MDR] when (...) you're not constantly describing the same thing over and over and over. [3MDR] was literally like ‘walk me through the memories'.”* (P7;T2.1)

“*In [3MDR] it's either you're not looking at it, and somebody saying ‘[P6] look at the screen'. That was huge. That was huge for me. When somebody says ‘you're avoiding this', like ‘no I'm not!' But then somebody actually shows you that you are not doing what you're supposed to be doing right now. Basically that's what [the therapist] said. In a nice way, but an honest way.”* (P6;T5)

“*You have a lot to do with that, your demeanor, the kind of person you are makes me feel comfortable, I could tell you everything, which is not necessarily who I am. But you managed to give me that comfortable feeling, a sense that I'm going to be all right… You tried to guess something about me that I don't think was 100%, but good on ya for exploring different avenues for me in my head.”* (P13;T4)

“*I think if you had the same person throughout, it would be better. More uniformity. Everything is so regimented—the same person is with you throughout. [With a new therapist] it's almost a different path or starting over. The new clinician doesn't know what buttons to push.”* (P10;T3)

##### “Breaking Through”: Opening the Vault

3MDR synergistically “cracked open” participants' internal vaults and broke down defenses, allowing participants to access, explore and attribute new meaning to trauma. With each session, experiences were unraveled “*like Pandora's box … with a plethora of things you have to deal with”* (P19;T4). Trauma-related physiologic responses were juxtaposed against a sense of security: “*wearing the harness felt like we had plates (body armor) … [which] felt good”* (P7;T5). Similarly, short exposures, with a break between exposure sets, resulted in an ebbing and flowing of psychological distress, and a greater ability to tolerate the therapy. Self-selection of photos reportedly provided a sense of control over the session, and frequent communication of SUD scores helped gauge distress. A positive “shift” in PTSD-related symptoms occurred as memories were confronted: “*[I became] more comfortable with (...) facing [my trauma] with the whole memory as opposed to just the negative pieces”* (P10;T2.5). For those who underwent conventional TFTs and found limited success, 3MDR was “*better able to provoke responses that actually need to be provoked”* (P7;T3), leading to greater insight into “*what they actually had to face”* (P10;T3). For some, this included addressing a range of feelings (e.g., guilt, shame, betrayal, anger, loss, rage) related to PTSD and MI.

“*When you go in, you have no idea what you are going into. It's hard and it hurts… The hard part for me was being on the platform and having that door open (on screen), [along with] the homework and trying to make sense of and dealing with the fallout. It's like opening that door and it's a flood gate: you can't close that door once it's open.”* (P19;T4)

“*I think the biggest stride came in week 4, when I smashed through that wall and really hit a low, an all-time low, and started to process that low and figure out why. I was there and things started to make sense and fall into place. That is the big thing: once you figure it out. Nobody can do that for you, you gotta figure it out. Once you figure it out, it's a lot easier to move forward.”* (P13;T4)

“*When we do the sessions we actually bring [the traumatic memory] up. Just the awareness itself is processing it in some manner. To speak of it, admit it to yourself, tell somebody else about it, on my own time. Then I go through the session and the words [are] used, and why and how they are associated. I will sit by myself and sort of think about the whole thing and what I said and how I actually feel about it, and take my time. Why that particular feeling... If I brought it up at home, I wouldn't think about the feelings. Just like reactions.”* (P10;T2.4)

#### Theme 2: Perceived Outcomes of 3MDR

While 3MDR treatment was often intense and difficult, it also enhanced participants' emotional regulation, sense of self and hope for recovery. Sub-themes capturing the depth of the participant's experience include: “Breaking Out of the Tomb,” “Things Are Lining Up,” and “A Return to Life.”

##### “Breaking Out of the Tomb”: I Can Finally Move

Participants described holding back traumatic memories, frequently leaving them feeling entombed within themselves until traumatic memories were processed. Entombment was accompanied by physical manifestations: “*For 20 years, I thought I was having a heart attack. At some point during the day, my heart would pound out of my chest, and I'd get tingly all over and break out in a sweat”* (P6;T5); another described, “*[C]razy knots; my muscles are so tense. I've got these knots all over my head and my jaw, and I clench, and I grind, and I strain”* (P5;T3). As traumas were addressed, their experiences changed: “*Waking up without chest pains… To have anxiety without the chest pain was a first. First time in 25 years”* (P10;T2.3). As burdens lifted, things started “*[G]etting a little brighter”* (P6;T5), and participants noted changes in their physical, psychological and emotional health. Most importantly, they began to feel reconnected to who they were prior to the trauma.

“*[S]essions have had a huge positive impact on me. They have allowed me to get a little more comfortable in my own skin, get a little less critical of myself, both from what happened there, and even what happens daily.”* (P11;T6)

“*There was a lot of garbage on top of who I was. The way I looked at it was there was this battle of you know the real me (…) and all of this crap that was on top of it…once I started offloading some of that junk yeah I guess things are getting a little brighter.. as more of me is being allowed out.”* (P6;T5)

“*I don't feel trapped [anymore] and that's a big thing. I felt trapped in my own little existence (…) Like ‘shit, this is the way it's going to be from now on' until whenever it ends, you know? And that was scary…very depressing. Like trapped. I was stuck there like ‘holy shit… I'm going to have to put up with this all the time, and eventually it's going to crush me'. But now I know that it doesn't have to be that way.”* (P6;T5)

“*Has [3MDR] changed my core? Maybe I'm getting back to my core.. [M]ore of me is being allowed out.” (P6;T5)*

##### “Things Are Lining Up”: Developing Emotional Awareness and Regulation

As participants processed traumatic memories, they reported gaining insight into distressing experiences and emotions and being better able to self-regulate. Participants gradually felt, “*[M]ore connected to my emotions”*, “*[L]earned to put words to feelings”* (P5;T2.6), and experienced, “*[L]ess urgency, less anxiety, less fear, less irritability”* (P5;T3). One participant described no longer having, “*the thoughts, images, memories slam into my brain, taking over the emotional aspect of things”* (P19;T5). Participants were increasingly able to contextualize their experiences and address core issues: “*[I]t's a complete 180 in perspective, I am no longer focused on trying to deal with anger and whatever. I am focused on the guilt and shame that caused the anger to begin with. I have gotten to an underlying layer of things… helped me realize it was real, to look at it in a different manner. Shift from anger to shame and guilt which is actually what I had to face. And I had never faced it until now”* (P10;T3). Simply, PTSD symptoms and strong emotional reactions significantly diminished over the course of 3MDR.

“*[T]he pictures were easier to face, the recovery was quicker. More and more each time… things just seemed to get easier.”* (P10;T2.4)

“*I began to forgive myself for my weakness. I didn't know what the issue was when I started 3MDR. All I knew was a very angry person. I came back from Afghanistan very very angry.”* (P13;T4)

“*What I have seen: a decrease in anxiety and general sort of (…) feeling down. Definitely a decrease in that ‘hide out in my room and zone out'. Since [3MDR], [P2] has shown a ton more motivation to do stuff. Working on the car, hanging out with [child] (…) picking up clothes on the floor. The overall motivation to do life.”* (P2;T3)

“*It helped in the fact that not only was I looking back remembering things and facing [the memories], but I was able to see some humor in them, not just the negative.”* (P10;T2.5)

##### “A Return to Life”: A Life Worth Living

Changes in attitude, outlook and relationships were commonly cited as treatment progressed. Participants became more able to confront the trauma on their own terms: “*[D]oing my own therapy. I am doing the stuff that I have to deal with”* (P2;T2.4). Processing traumatic memories enabled them to experience greater confidence, hope, and possibility: “*This whole intervention thing has really shown me that it is possible for me to move on from what happened 10 years ago”* (P7;T5). While scars of memories remained, raw, deeply-seated wounds began to mend: “*It's almost like stitches. Still a wound, going to leave a mark, but a healing process”* (P19;T3). As trauma was processed, motivation improved and participants were able to more confidently turn their attention to the present and future (e.g., some participants described reconnecting with themselves and their families).

“*[The change] is in my body, it's in my... everything. 100%. It's been a change in my circle … my whole life...changed me physiologically, changed my family.”* (P6;T5)

“*I've reached out to some people that I haven't talked to in a long time… it was good. (...) The guys are happy to hear from me, and happy to hear that I'm alive.”* (P7;T5)

“*My focus has improved, concentration has improved. Go to work, and do paperwork. I still tire quickly when I have to pay attention to details; it does wear me out, but at least I can do it now. Where before I wouldn't have been able to… improvement is there but it's not 100%.”* (P2;T3)

“*I kinda lost who I was [before 3MDR]. It's kinda hard for me to see who I really was before all this happened, but once I started getting rid of that crap, I guess I started finding myself again… I think I'm really getting back to who I was.”* (P6;T5)

“*I'd say I'm more active [post-3MDR]. Just trying to get out and go to the gym more and try to get back to being a functioning member of society I guess. This might have been the kick that I needed to get off this ‘pity party train' that I've been having.”* (P7;T5)

#### Theme 3: Keys to Successful 3MDR Experiences

Participant experiences with 3MDR offer insights into salient aspects of the therapeutic process and ingredients for success. These are reflected in the following sub-themes: “Timing and Readiness Matters” and “The Person is Central”.

##### “Timing and Readiness Matters”

Participant readiness for 3MDR and timing of engagement were important factors impacting treatment effectiveness. Prior experience with TFTs readied some 3MDR participants by familiarizing them with therapeutic modalities and equipping them with language and coping strategies. Regarding timing, one participant commented, “*I don't know if this is something that you want to expose people to immediately after their trauma”* (P7;T2.3); 3MDR possibly, “*[W]ould be more beneficial for people who have left the service”* (P13;T4). “*If I was still serving… I would still want to be that person… I think it would have been tougher. Now that I'm a civilian, not having to worry about your troops (...). Whatever is inside gnawing at them, it's not who they were anymore. You did the job, but now you have moved on. You got sneakers on instead of combat boots”* (P13;T4). Alternatively, one Veteran postulated, “*If I had done [3MDR] after my first or second tour, the [recovery] time would have been shorter”* (P2;T4b). While more immediate access to 3MDR may be helpful, participants indicated that active duty MMs may be reluctant to seek it over concerns around perceived operational readiness or career implications.

“*[3MDR] can give me all the tools in the world, spend millions of dollars on [the intervention], but at the end of the day, if I don't come to the table with that willingness to confront the issues, [this therapy] won't be effective.”* (P7;T2.2)

“*Week 5 [was the turning point because of] the avoidance thing too. If I had done [3MDR] earlier, I definitely would have reaped more benefits.”* (P6;T2.6)

“*Definitely more open to it, at first I thought I will try anything once. But now I see it has potential, I should give it an honest shot.”* (P10;T2.1)

##### “The Person Is Central”: Tailored Immersion

Personalization of 3MDR facilitated through tailored immersion (e.g., determination of traumatic memories, selection of photos and music), participant-therapist fit, treatment dose, and meaningful debriefs were essential. Critically important was participant empowerment: “*I dictate the narrative (...). If it gets too much I can say stop. 90% of it I am in control of; they facilitate. I decide the speed on the treadmill, I pick the pictures, all my work, it's all… me. It's just facilitated by other people”* (P2;T4c). Participant-therapist fit required consideration of the person and professional training of the therapist, as well as their military affiliation, the latter of which, while “*not necessarily the most important connection”* (P6;T5), was facilitative for some, and an impediment for others: “*I get that he's walked the walk, but I think I want someone who doesn't understand… I've already been with people who understood, and they've been dismissive”* (P11;T3). Regarding dosing, participants may require variable numbers of sessions: “*[At sessions] three and four, you are starting to just scratch at what the big issues are”, [and if limited to six], “How are you supposed to get a treatment to latch and be effective?”* (P2;T3). Equally, reflective debriefing facilitated reconsolidation and safety.

“*[Therapists] were always really concerned at the end, like making sure I was okay (...), I really appreciated that. I've had therapists in the past where it's like ‘okay, you just had a monumental session and it's clearly very distressing to you, but we're 15 minutes overdue for my next session so you need to [leave]…', that's (...) why I stopped going to therapy.”* (P7;T3)

“*I know at least one of the pictures I don't think I'm done with (…) Maybe I'll change my pictures for next time so we're dealing with that one a couple of times instead of just once.”* (P5;T2.5)

“*I had real difficulty with a military guy beside me, because I don't want to be around it anymore (…) I get the concept that he's walked the walk, but I think I want someone who doesn't understand (…) I've already been with people who understood, and they've been dismissive.”* (P11;T3).

“*The trick is, someone has to be able to relate to the individual on [the 3MDR system]. For me, I couldn't be on that machine with someone who has never seen military life, or war or [has] never been on operation. It wouldn't work. If you were [a cop] and on [the treadmill] with a 25-year-old social worker who had just finished school, what would your immediate feeling be? He or she would be talking and you would be like ‘shut up', because you have no idea.”* (P2;T4a)

## Discussion

This paper uniquely utilizes a qualitative thematic analysis to explore the experiences of MMs and Veterans with TR-PTSD as they engaged in 3MDR. The results of this paper contribute to and complement existing quantitative publications on 3MDR through the participant perspective during and after the intervention. Three main themes emerged from the data: (1) the participants' experiences with 3MDR; (2) perceived outcomes of 3MDR; and (3) keys to successful 3MDR treatment. Overall, the participants' attributed the effectiveness of 3MDR to the personalized multimodal environment, including walking and dual attention tasks, and the therapeutic alliance which, when combined, prevented disengagement from the experience.

This attribution is similar to the theoretical underpinnings being proposed by current 3MDR research demonstrating the combination of immersive virtual reality, physical movement, and the collaborative therapeutic relationship as facilitators of 3MDR's effectiveness. Participants felt the intense exposure-based environment was central to the perceived effectiveness of 3MDR. These experiences are complemented by current literature demonstrating that direct interactions with traumatic stimuli within exposure-based therapies is essential to the reduction of PTSD symptoms and breaking patterns of avoidance (34). While the underlying mechanisms of 3MDR are still being explored, research suggests that 3MDR limits the ability of participants to avoid trauma-related cues (20, 25). The increasing emotional intensity of sequential images within 3MDR appears to minimize avoidance. Participants noted that viewing the images allowed them to process distressing emotions and facilitate improvements in their emotional regulation during the treatment by enabling participants to adaptively respond to negative emotions arising from threatening or aversive stimuli (35).

Our findings complement the growing body of evidence suggesting 3MDR may effectively address emotional regulation by providing a healthy way for participants to remain grounded and connected while processing distressing emotions (28, 29, 35). Difficulties with emotional regulation are common in PTSD, with emotional dysregulation being positively associated with PTSD severity (36) and maintenance of PTSD symptoms (37, 38). Addressing emotion may be as important as addressing the fear-based stimuli (39). Study results add to preliminary quantitative results from a 3MDR randomized control trial showing an increase in emotional regulation following 3MDR (35, 40). Within the current study, participants felt that “breaking through” and reconsolidating traumatic memories allowed them to reconnect with their “lost selves” and provided hope for recovery.

The quality of the client-therapist alliance is significant as it relates to PTSD treatment outcomes (41). Participants felt 3MDR sessions were emotionally strenuous and activating, and feeling supported was frequently regarded as an essential component. During 3MDR, participants reported strong connections with their therapists, who provided a sense of safety and reassurance. Participants in our study reported experiencing a non-judgmental connection to their therapist via small acts, which may have enhanced the participant's engagement and ongoing participation in therapy (42). Participants felt changes in therapists mid-treatment affected their ability to trust and exhibit vulnerabilities which, in turn, reduced the effectiveness of the treatment. This is supported by literature indicating reduced PTSD treatment outcomes following unresolved breaks in the therapist-client alliance (43).

The ambulatory component of 3MDR was often cited by participants as a perceived mechanism of the treatment's success. Participants indicated that “walking through” traumatic images in 3MDR assisted them. This participant experience may be supported by embodied cognition theory, which hypothesizes that physical actions influence both perception and cognition: perception is followed by cognition, which is followed by action (44). Embodied cognition theory may explain how human cognitive processes are shaped by sensorimotor information (e.g., posture, movement and internal bodily states), which is supported by participant reports within this study. As an example, participants in our study reported actively facing trauma physically, emotionally and symbolically with movement contrasted against previous experiences of being stagnant while seated in a traditional therapy chair. Walking may also facilitate divergent thinking, which allows for greater cognitive flexibility and the ability to perceive traumatic narratives and meaning in a different way (45). Although the neurological mechanisms for how movement can assist with trauma therapy require further exploration, the participants expressed that this evolution from attaining therapy in a static position to a dynamic, ambulatory experience greatly improved the effectiveness of 3MDR.

While much more is yet to be learned about participant experiences of 3MDR, the insights gained lay a foundation for further research leading to 3MDR's potential future real-world application as a recognized evidence-based intervention.

### Implications for Future Use of 3MDR With Trauma-Affected Populations

Research is demonstrating that 3MDR has the potential to treat MMs and Veterans with TR-PTSD, paving the way for its use in real-world clinical contexts with trauma-affected populations. Several key factors, however, yet require consideration. First, the populations with which 3MDR can be used and the timing of engagement are important. An individual's state of readiness and possible negative career and relational impacts can all affect an individual's acceptance of 3MDR. Second, identification and management of symptom exacerbation are essential given that some participants report a temporary increase in symptoms before experiencing benefits. Third, training healthcare professionals to effectively deliver 3MDR to specific populations is paramount.

## Future Research

Future research into 3MDR is warranted, including its underlying mechanisms, appropriate populations, and optimal preparation, the timing of delivery and dosing. Whether 3MDR is effective at addressing the co-occurrence of PTSD and MI in MMs and Veterans with TR-PTSD also warrants consideration and is the subject of an upcoming analysis by the research team. Delivery formats, including in-person and augmented reality, and training of new therapists, also require future study.

## Strengths and Limitations

This study has numerous strengths and limitations. Most notably, this qualitative analysis is one of few studies that report on the experience and impact of 3MDR on MMs and Veterans with TR-PTSD [see also (20)]. The use of rigorous qualitative methodology by a multidisciplinary team strengthened findings. Regarding limitations, the sample size for this pilot analysis (*n* = 13) which was small due to restrictions associated with the COVID-19 pandemic, is not representative of all MMs and Veterans and cannot be overgeneralized; results from the full study will be forthcoming. Further, the quality of recordings and missing data may have impacted the accuracy of analysis, and some participants chose not to participate in all of the data collection resulting in missing longitudinal data.

## Conclusion

MMs and Veterans are at risk of developing TR-PTSD and moral injury, with worse treatment outcomes than in civilians. First-line TFTs have limitations among which are cognitive avoidance and high dropout rates. 3MDR aims to overcome these by active engagement and empowerment. Results of this qualitative thematic analysis contribute to the evidence-base regarding 3MDR and indicate that it shows promise as an effective intervention for TR-PTSD. Participants reported significant and sustained functional, well-being and relational improvements upon treatment completion. Key to helping participants attribute new meaning to a traumatic experience is 3MDR's multi-modal nature, participants' control of their therapy, tailored immersion, and role of the therapist as coach and fire team partner. Exploration of the use of 3MDR with other trauma-affected populations in real-world settings is warranted.

## Data Availability Statement

The original contributions presented in the study are included in the article/supplementary material, further inquiries can be directed to the corresponding author.

## Ethics Statement

The study was conducted according to the guidelines of the Declaration of Helsinki and approved by the University of Alberta Research Ethics Board (Pro00084466, 21 January 2019) and the Canadian Armed Forces (CAF) Surgeon General Research Program (E2019-02-250-003-0003, 1 April 2019).

## Author Contributions

SB-P, CJ, and EV conceived, designed, and conducted the study. All authors drafted, revised, and approved the final manuscript submitted for publication.

## Funding

This research was funded by the Government of Canada Innovation for Defence Excellence and Security (IDEaS) (grant number CPCA-0617); the Government of Alberta (grant number 011427); the Glenrose Rehabilitation Hospital Foundation (grant number RES0042203); and the Royal Canadian Legion (grant numbers RES0048730 and RES0046384). In-kind support was provided by Alberta Health Services, Veteran Affairs Canada, the Canadian Armed Forces, and the academic institutions of which the research team members are affiliated.

## Conflict of Interest

The authors declare that the research was conducted in the absence of any commercial or financial relationships that could be construed as a potential conflict of interest.

## Publisher's Note

All claims expressed in this article are solely those of the authors and do not necessarily represent those of their affiliated organizations, or those of the publisher, the editors and the reviewers. Any product that may be evaluated in this article, or claim that may be made by its manufacturer, is not guaranteed or endorsed by the publisher.
